# Molecular Pathways of the Therapeutic Effects of Ayahuasca, a Botanical Psychedelic and Potential Rapid-Acting Antidepressant

**DOI:** 10.3390/biom12111618

**Published:** 2022-11-02

**Authors:** Giordano Novak Rossi, Lorena T. L. Guerra, Glen B. Baker, Serdar M. Dursun, José Carlos Bouso Saiz, Jaime E. C. Hallak, Rafael G. dos Santos

**Affiliations:** 1 Department of Neurosciences and Behavior, Ribeirão Preto Medical School, University of São Paulo, Ribeirão Preto 14040-900, Brazil; 2 National Institute of Science and Technology—Translational Medicine, Ribeirão Preto 3900, Brazil; 3 Department of Psychiatry (Neurochemical Research Unit) and Neuroscience & Mental Health Institute, University of Alberta, Edmonton, AB T6G 2G3, Canada; 4 ICEERS Foundation, International Center for Ethnobotanical Education, Research and Services, 08015 Barcelona, Spain; 5 Medical Anthropology Research Center (MARC), Universitat Rovira i Virgili, 43001 Tarragona, Spain; 6 Departamento de Neurociências e Ciências do Comportamento, Faculdade de Medicina de Ribeirão Preto, Universidade de São Paulo, Hospital das Clínicas, Terceiro Andar, Av. Bandeirantes, Ribeirão Preto 3900, Brazil

**Keywords:** ayahuasca, DMT, antidepressant, psychedelic

## Abstract

Ayahuasca is a psychoactive brew traditionally used in indigenous and religious rituals and ceremonies in South America for its therapeutic, psychedelic, and entheogenic effects. It is usually prepared by lengthy boiling of the leaves of the bush *Psychotria viridis* and the mashed stalks of the vine *Banisteriopsis caapi* in water. The former contains the classical psychedelic N,N-dimethyltryptamine (DMT), which is thought to be the main psychoactive alkaloid present in the brew. The latter serves as a source for β-carbolines, known for their monoamine oxidase-inhibiting (MAOI) properties. Recent preliminary research has provided encouraging results investigating ayahuasca’s therapeutic potential, especially regarding its antidepressant effects. On a molecular level, pre-clinical and clinical evidence points to a complex pharmacological profile conveyed by the brew, including modulation of serotoninergic, glutamatergic, dopaminergic, and endocannabinoid systems. Its substances also interact with the vesicular monoamine transporter (VMAT), trace amine-associated receptor 1 (TAAR1), and sigma-1 receptors. Furthermore, ayahuasca’s components also seem to modulate levels of inflammatory and neurotrophic factors beneficially. On a biological level, this translates into neuroprotective and neuroplastic effects. Here we review the current knowledge regarding these molecular interactions and how they relate to the possible antidepressant effects ayahuasca seems to produce.

## 1. Introduction

Ayahuasca is a psychedelic preparation usually made by the decoction of *Banisteriopsis caapi* and *Psychotria viridis*, or *Diplopterys cabrerana*, plants endemic to the Amazonian Basin where the brew is traditionally used in ritualistic contexts [1,2,3]. *B. caapi* is known to contain a class of substances called β-carbolines or harmala alkaloids, mainly harmine, tetrahydroharmine (THH), and harmaline [4,5]. These substances are known to selectively and reversibly inhibit the enzyme monoamine oxidase type A (MAO-A), which is believed to be their main mechanism of action [5,6]. On the other hand, *P. viridis* is a source of DMT, a serotoninergic psychedelic belonging to the same pharmacological class of substances as lysergic acid diethylamide (LSD) and psilocybin [7]. The main mechanism of action for DMT and related psychedelic substances is widely accepted to be agonism at the serotonin receptors 5-HT_1A,2A,2C_, with the 2A subtype being the primary molecular target and its activation dose-dependently related to the psychoactive effects these substances cause [8,9,10,11]. In the case of ayahuasca, the presence of β-carbolines inhibits the main DMT metabolic pathway via MAO-A inactivation, which in turn allows for DMT to be orally active [11,12,13].

In the last several decades, scientific research on ayahuasca consumption and administration has been conducted to investigate its possible therapeutic effects. From pre-clinical investigations [14,15,16,17,18,19,20,21,22] to observational studies [13,23,24,25,26,27,28,29,30,31,32,33], clinical trials [11,34,35,36,37,38,39,40,41], reviews, and meta-analyses [2,42,43,44,45,46,47,48,49,50], there is accumulating evidence that ayahuasca and its isolated substances may present neuroprotective, neuroplastic, immunomodulatory, anticancer, anti-addictive, anxiolytic, and, most notably, antidepressant properties, while also increasing well-being and quality of life and improving emotion regulation. Investigations on the therapeutic properties of psychedelics are exploring possibilities to use these substances for treating several psychiatric disorders, especially depression [43,46,51]. Clinical evidence already points to possible antidepressant and anxiolytic properties in psilocybin, LSD, ayahuasca, and DMT [38,43,52,53]. Additionally, ketamine, a non-classic psychedelic, has recently been approved as an alternative medication for treatment-resistant depression [54]. 

Besides the most prominent mechanisms of action cited above, ayahuasca’s substances seem to act in synergy when consumed together, directly or indirectly modulating many other molecular targets which may include not only serotoninergic receptors but also glutamatergic [55,56], dopaminergic [57,58], and endocannabinoid [35,59] systems, the serotonin transporter (SERT) [60,61], the VMAT [60], the TAAR [62], and the sigma-1 receptor [63,64]. With this complex pharmacological profile [65] in evidence, this review sought to analyze the molecular targets of ayahuasca and its substances and relate their modulation to the possible therapeutic effects the brew seems to provide.

## 2. A Brief Review of the Current Evidence for the Antidepressant Effects of Ayahuasca in Humans

As with other classical psychedelics such as psilocybin and LSD, published research regarding the therapeutic effects of ayahuasca has grown steadily in the last two decades. Although preliminary and still lacking further and more thorough evaluation, results so far have been promising, especially regarding the antidepressant and anxiolytic effects the brew seems to exert. Grob et al. (1996) [66] performed the first observational study with frequent ritual ayahuasca practitioners that reported possible antidepressant effects of the brew. Similar results have been demonstrated in observational studies in the following years by other groups [24,29,32,67,68]. The first preliminary open-label clinical trial investigating the antidepressant effects of ayahuasca was published by our group in 2015 [37], which was followed by a complementary study that expanded the sample in 2016 [40]. The first randomized, placebo-controlled trial (RCT) investigating the antidepressant effects of ayahuasca replicated the positive results found in the preliminary clinical trial [38]. Nevertheless, small sample sizes, single-dose administration, short time assessments, and alkaloid dose standardization are limitations that still need to be addressed in future research on the topic. Table 1 below summarizes the current evidence regarding ayahuasca’s antidepressant effects in depressed patients, which was basically derived from two clinical trials (one open and one placebo-controlled).

## 3. Safety and Tolerability of Ayahuasca Administration

Although there is no formal report of deaths caused by ayahuasca intake, the lack of regulation over its production and consumption in some countries can result in medical tourism scenarios [72]. Excessive optimism of psychedelics’ therapeutic properties portraited in mediatic coverage can mask the consequences of reckless use, namely interaction with other psychoactive substances (such as Selective Serotonin Reuptake Inhibitors (SSRIs)) and with previous medical conditions, which can produce lifelong health impairments [72,73].

Ayahuasca consumption at reported doses in observational studies and clinical trials causes a wide variety of effects, of which some are desired, some are unwanted, and/or can be considered adverse events (AEs). Regarding randomized, blinded (single or double), and placebo-controlled trials, a recent review evaluated the occurrence of adverse events following ayahuasca administration to healthy volunteers and treatment-resistant depressive patients in 11 distinct trials (*n* = 108 ayahuasca administrations) [74]. On one hand, most common AEs reported were gastrointestinal malaise, nausea, vomiting, headaches, and mild-to-moderate transient increases in heart rate and blood pressure [74]. These AEs were all expected and known to occur with ayahuasca and other psychedelic administration such as LSD and psilocybin [74,75]. On the other hand, there were more clinically significant reports of anxiety, confusion, emotional distress, depersonalization, and dysphoric state manifestations after ayahuasca administration, although these are much less common. However, there are currently no reports of psychotic states, lasting AEs, trial dropouts, or the need for medical interventions in any case, even in more significant situations. All reported AEs were transient and resolved on their own with researcher’s psychological support [74].

Regarding the occurrence of more significant adverse events in clinical trials with ayahuasca performed by our group, there is a detailed report of the two instances where these effects had to be managed by our research team, how the situation was resolved, and an initial nine step guideline on managing this kind of occurrence [76]. Overall, it seems that as with other psychedelic administration, a cautious and detailed selection of the volunteers who are allowed to participate in the trials and a supportive setting constructed by researchers has been demonstrated to be enough to reduce and manage the occurrence of AEs in clinical trials. Nevertheless, there is still the need for further research with bigger samples to confirm the preliminary findings we have so far for the occurrence of both positive and negative effects.

## 4. Ayahuasca’s Alkaloid Content

The concentrations of psychoactive alkaloids in ayahuasca vary greatly due to the lack of standardization in the quantity and quality of the plants used in its production, the region where it is produced, cultural aspects related to its use, and the desired final concentration of the drink [77]. Previous studies that quantified the alkaloid levels present in diverse ayahuasca samples reported a wide range of concentrations. For example, the content of alkaloids reported by McKenna et al. (1984) [5] ranged from 0.15 mg/mL of harmine, 0.05 mg/mL of THH, and 0.125 mg/mL of DMT, to 4.67 mg/mL of harmine, 1.60 mg/mL of THH, 0.41 mg/mL of harmaline, and 0.60 mg/mL of DMT in a dose. Callaway (2005) [78] measured the levels of alkaloids in 29 samples of ayahuasca from different religions where it is consumed. Again, a wide variation in alkaloid levels was demonstrated in the different samples, with DMT ranging from 0 to 14.15 mg/mL, harmine from 0.45 to 22.85 mg/mL, THH from 0.48 to 23.8 mg/mL, and harmaline from <0.01 to 0.9 mg/mL [78]. Santos et al. (2017) [79] validated a solid-phase extraction technique to quantify the alkaloids of 20 ayahuasca samples, where the levels of DMT, harmine, harmaline, THH, tryptamine, and harmalol were evaluated in concentrations ranging from 0.3 to 36,7 mg/ml. Souza et al. (2019) [80] quantified DMT, harmine, THH, and harmaline in 38 ayahuasca samples. DMT was reported in concentrations from 0.62 to 3.4 mg/mL, harmine from 4.14 to 18.16 mg/mL, THH from 4.02 to 30.88 mg/mL, and for harmaline from 0.4 to 3.92 mg/mL. Table 2 below summarizes the data for studies that evaluated alkaloid levels in at least eight ayahuasca samples.

Even across clinical trials with healthy and depressed patients, there is still a lack of standardization of the dosage administered to participants. Table 3 below illustrates this variation.

This variation in alkaloid administration in investigations where ayahuasca is used is one of the major challenges associated with its possible use as an antidepressant. Although clinical research with ayahuasca is still in its infancy, the necessity for standardizing dosages given in different trials is becoming more apparent. In the current state, it is difficult to directly compare results amongst the published research. Furthermore, with the current published data it is very challenging to determine the optimal DMT to β-carboline ratio and dosages that maximize the therapeutic effects while also minimizing the occurrence of AEs. This and other challenges associated with standardized ayahuasca medicinal use (and other psychedelics) go beyond the main focus of this article and have been thoroughly discussed elsewhere **[74,75,76,87]**.

## 5. Pharmacokinetics of Ayahuasca

The human pharmacokinetics of ayahuasca were evaluated for the first time in a study by Callaway et al. (1999) [88] after the administration of 2 mL/kg of ayahuasca (with alkaloid concentrations: harmine 1.70 mg/mL, harmaline 0.20 mg/mL, THH 1.07 mg/mL, and DMT 0.24 mg/mL). In this study, the following values of maximum concentration (C_MAX_) and time to reach C_MAX_ (T_MAX_) were found in blood analysis: DMT—C_MAX_ 15.8 ± 4.4 ng/mL, T_MAX_ 107.5 ± 32.5 min; harmine—C_MAX_ 114.8 ± 61.7 ng/mL, T_MAX_ 102.0 ± 58.3 min; THH—C_MAX_ 91.0 ± 22.0 ng/mL, T_MAX_ 174.0 ± 39.6 min; harmaline—C_MAX_ 6.3 ± 3.1 ng/mL, T_MAX_ 145.0 ± 66.9 min. Another investigation looking at plasma alkaloid levels after administration of a low dose (standardized at 0.6 mg/kg DMT) and a high dose (standardized at 0.85 mg/kg DMT) of lyophilized ayahuasca was performed by Riba et al. (2003) [11]. In this study, C_MAX_ values were found for DMT of 12.14 ng/mL and 17.44 ng/mL for low and high doses, respectively, with T_MAX_ of 1.5 hours after administration for both doses. Dos Santos et al. (2011) [1] measured plasma DMT levels after administration of placebo, one dose and two consecutive doses of ayahuasca four hours apart, at a standardized dose of 0.75 mg/kg DMT. The mean ± SD of maximum concentration values was 13.97 ± 9.35 ng/mL for ayahuasca preceded by placebo and 32.57 ± 20.96 ng/mL for ayahuasca preceded by ayahuasca, suggesting a non-linear increase in DMT levels after administration of two consecutive doses of ayahuasca, likely due to prolonged peripheral MAO-A inhibition [1]. Another study by the same research group demonstrated a comparison with a single dose of ayahuasca (standardized at 1 mg/kg of DMT) and 20 mg of dextroamphetamine [84]. The mean ± SD of maximum plasma DMT concentration values was 11.8 ± 6.4 ng/mL. The median time at which C_MAX_ was reached was 1.8 hours (range 1 to 4.5 hours) after administration [84].

According to these data, we can estimate the nM levels of the alkaloids at C_MAX._ Considering a single ayahuasca administration, the mean C_MAX_ for DMT of the cited studies where C_MAX_ was measured was (15.8 + 12.14 + 17.44 + 13.97 + 11.8)/5 = 14.23 ng/mL. The molar mass of DMT is 188.274, which results in a 75.58 nM mean maximum plasma concentration. At first this concentration appears to be lower than the required to interact with certain molecular targets, but there is an evidence-based proposed mechanism through which DMT can reach high local concentrations within neurons of the CNS [60,89]. Briefly, this three-step mechanism starts with the crossing of DMT through the blood-brain barrier (BBB) via uptake across the endothelial plasma membrane. To cross the BBB, DMT is actively transported through the endothelial plasma membrane via Mg^2+^ and ATP-dependent uptake [90,91,92]. Although we are not aware of an investigation regarding the precise mechanism by which this accomplished, it is possible that the Organic Cation Transporter (OTC) family of transporters is involved, since it can transport other closely related monoamines, such as serotonin [93]. 

This is corroborated to happen given the results from studies that verified the accumulation of DMT and other tryptamines in the brain after peripheral administration [91,94,95,96]. It has been shown that DMT promptly enters the CNS and is kept there after excretion in urine has stopped (24 hours), being detected up to 7 days after administration [97]. Next, cell-membrane SERT uptakes DMT to inside the neurons, where VMAT2 promotes its sequestration and accumulation within vesicles [89]. Here DMT is protected from MAO degradation and can be stored several days before being released when the correct stimuli are given [97]. The proposed mechanism and investigations that support it are evidence of DMT’s role as an important cellular messenger, since there is a considerable physiological effort and prioritization to transport, accumulate, and store it within the CNS [89]. Furthermore, it shows that DMT must have molecular activity endogenously in a much lower average concentration environment inside the human body, in the absence of exogenous intake [89]. If this molecule is active within this relatively hostile environment to its existence, exogenously administered DMT in tandem with peripheral degradation inhibition provided by MAO-A deactivation from β-carbolines surely augments its concentrations to levels high enough to influence other molecular targets it would otherwise not be capable of influencing. The current evidence for the action of DMT and β-carbolines within these possible targets is discussed with more detail in the next sections.

## 6. Ayahuasca’s Molecular Targets and Their Relation to Possible Antidepressant Effects

**Figure 1:**
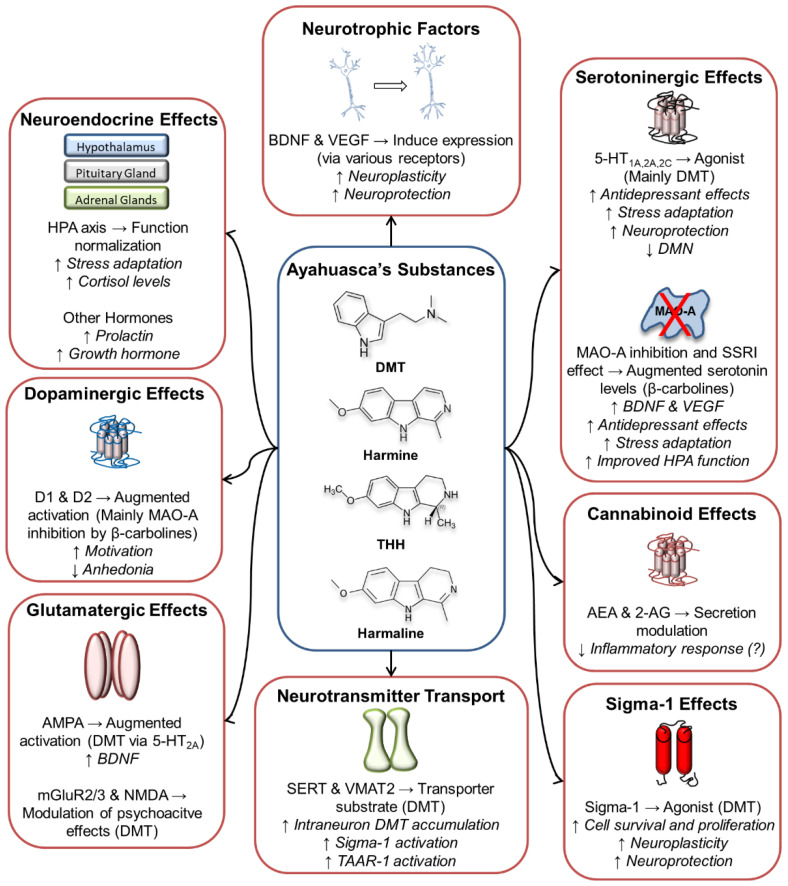
Main molecular targets and effects of ayahuasca’s alkaloids. 2-AG: 2-Arachidonoylglycerol; 5-HT: Serotonin; AEA: Anandamide; AMPA: α-Amino-3-hydroxy-5-methyl-4-isoxazolepropionic acid receptor; BDNF: Brain-Derived Neurotrophic Factor; D1 and D2: Dopamine receptors 1 and 2; DMN: Default Mode Network; DMT: N,N-dimethyltryptamine; HPA axis: Hypothalamic-Pituitary-Adrenal axis; mGluR: Metabotropic Glutamate Receptor; MAO-A: Monoamine Oxidase A; NMDA: N-Methyl-D-aspartate receptor; SERT: Serotonin Transporter SSRI: Selective Serotonin Reuptake Inhibitor; TAAR-1: Trace Amine-Associated Receptor 1; THH: Tetrahydroharmine; VEGF: Vascular Endothelial Growth Factor; VMAT2: Vesicular Monoamine Transporter 2.

The following paragraphs will focus on the molecular targets of ayahuasca and its constituent substances in several neurotransmission systems. Figure 1 below summarizes the main effects described.

### 6.1. Serotoninergic System

As the main target of action of DMT and modulated by MAO-A inhibition via β-carbolines, the serotoninergic system is greatly affected by the ingestion of ayahuasca. DMT binds to multiple serotonergic receptor subtypes including 5-HT_1A,1B,1D,2A,2B,2C,5A,6,7_ with varying degrees of affinity [98,99,100,101,102,103] where it is believed to act as a partial or full agonist [102,104]. This system has been a primary target for antidepressant drugs for many decades, with MAO-inhibiting drugs being first introduced in the 1950s [105]. The β-carboline harmane has been shown to increase serotonin release in vitro [106], and acute peripheral harmane injection in rats resulted in increased levels of serotonin in the hippocampus, amygdala, the prefrontal cortex (PFC), and hypothalamus [107]. Furthermore, THH has been shown to possess an SSRI effect [108]. Considering many antidepressants and anxiolytic drugs act through similar mechanisms (i.e., increasing serotonin neurotransmission), the direct interaction with serotoninergic receptors by ayahuasca’s alkaloids and augmented levels of circulating serotonin via MAO-A inhibition are thought to be important protagonists in these possible antidepressant effects of the brew.

Although ayahuasca seems capable of influencing many serotonin receptors concomitantly, human evidence suggests that the 5-HT_2A_ receptor is responsible for most of the subjective and neurophysiological effects of its administration [109]. Furthermore, this has been corroborated by similar research with LSD [9,110,111] and psilocybin [112,113]. Furthermore, while other serotonin receptors besides 5-HT_1A,2A,2C_ may also influence ayahuasca’s effects, research that regards these receptors’ specific roles after DMT/β-carbolines/ayahuasca administration does not exist to the best of our knowledge. Taking these points in consideration, the next paragraphs will focus on the discussion of the activation of 5-HT_1A,2A,2C_ receptors.

The 5-HT_1A_ Gi/Go-protein coupled receptors are one of the most prominent targets for currently approved antidepressant drugs [114,115] and are believed to have a key role in the etiology and treatment of depression [116]. Whether through direct or indirect mechanisms, SSRIs, tricyclic antidepressants (TCAs), MAOI, other antidepressant drugs and therapies increase 5-HT_1A_ post-synaptic signaling [117,118,119,120]. Furthermore, the therapeutic effects of antidepressants have been related to the neurogenesis associated with the activation of this receptor [121,122].

These receptors are involved in the molecular cascades of phospholipase-C activity regulation, inhibition of cAMP accumulation, adenylyl cyclase inhibition, and calcium current reduction [114,123,124] and are abundant in the hippocampus, hypothalamus, amygdala, cingulate, and infralimbic cortex, raphe nuclei, and layers I-II and to a lesser extent V-VI of the cerebral cortex [115,116]. In cortico-limbic areas, 5-HT_1A_ receptors are located post-synaptically, while in the raphe nuclei (where the serotoninergic neurons project from) they are found as autoreceptors responsible for controlling serotonin release, activity, and neuronal firing to projected areas [117].

Ayahuasca’s alkaloids concomitantly modulate these receptors, with DMT being a 5-HT_1A_ agonist [125] and β-carbolines enhancing their activation via the SSRI effect of THH and augmented serotonin neurotransmission by inhibiting MAO-A-induced serotonin degradation, a process which also occurs with other MAO-inhibiting antidepressant drugs [126]. It has previously been shown that harmaline-induced hypothermia was attenuated by co-administration with a 5-HT_1A_ receptor antagonist, although the exact mechanism by which this occurs is not clear [127]. In humans, administration of pindolol (a 5-HT_1A_ antagonist) significantly increased psychological responses to i.v. DMT, suggesting a buffering effect of 5-HT1_A_ agonism on 5-HT_2A_-mediated effects [102]. Agonistic activation of this receptor has been proposed to improve stress adaptation [128,129,130], positively modulate the HPA axis [129,131], and possibly be related to neuroplastic effects [132,133], processes related to the remission of depressive symptoms. Preclinical behavioral investigations have shown that the deletion of 5-HT_1A_ heteroreceptors in the dentate gyrus granule cells of the hippocampus abolished the positive effects of SSRIs in various tasks and attenuated the neuroplastic effects associated with their administration via a reduction in the expression of the brain-derived neurotrophic factor (BDNF) and vascular endothelial growth factor (VEGF) [122], which further demonstrates their importance for treatment of depression. 

Arguably the most important molecular target for the effects of ayahuasca is the 5-HT_2A_ Gq/G11 protein-coupled receptor. Neurons expressing 5-HT_2A_ receptors are located mainly on layer V of pyramidal neurons from the neocortex, but also in limbic and basal ganglia structures such as the mammillary bodies of the hypothalamus, hippocampus, nucleus accumbens (NAc), amygdala, caudate, and putamen [134,135]. On a molecular level, the activation of these receptors induces intracellular signaling cascades which include phosphoinositide-specific phospholipase C (PLC)-induced increase in inositol 1,4,5-trisphosphate (IP3) and diacylglycerol (DAG), extracellular signal-regulated kinases (ERK), tyrosine kinase pathways, and a β-arrestin2-mediated pathway that results from serotonin binding [135,136,137,138]. DMT binding to these receptors activates the phospholipase A2 signal transduction pathway, leading to more arachidonic acid release and less inositol phosphate formation [104,139].While 5-HT_2A_ modulation by DMT is considered to be the main mechanism of action from ayahuasca, β-carbolines can also act as serotonin 5-HT_2A_ receptor agonists, although generally with less affinity (K_i_ 230 nM for harmine, 7780 nM for harmaline, and >10,000 nM for THH) [140,141]. 

Preclinical evidence has demonstrated that chronic daily treatment with ayahuasca (28 days) in rats resulted in increased hippocampal BDNF levels in females [142]. Corroborating these findings, results from an RCT where ayahuasca was administered to treatment-resistant depressive patients revealed higher serum BDNF levels 2 days after treatment in both patients and controls when compared with the placebo [71]. Activation of 5-HT_2A_ receptors has also been implicated in neuroprotective and neuroplastic effects [12,143]. It has been demonstrated that agonists at these receptors prevented NMDA antagonist neurotoxicity in the rat brain [144]. The activation of these receptors through administration of ayahuasca and other psychedelics has also been related to reduced activity of the default mode network (DMN), a brain-wide network that is overactive in depressed patients and related to rumination occurrence [145,146]. Indeed, there is evidence of neuroplastic effects in the PCC (a key node of the DMN) associated with long-term ritual ayahuasca use [25]. Ritual users showed increased cortical thinning in the PCC, which was inversely correlated with the intensity and duration of prior use of ayahuasca and with scores on the personality trait of self-transcendence. Users also showed increased cortical thickness in the ACC, a region prominent in the DMN that is related to emotional regulation and response to antidepressants [147].

Importantly, it is not yet known if the psychedelic effects induced by 5-HT_2A_ receptor activation are necessary for the antidepressant or other therapeutic effects to occur. Although there is pre-clinical evidence that they are not [148] and that ayahuasca acts through many other pathways to exert its effects, studies on the concomitant administration of 5-HT_2A_ receptor antagonists (such as ketanserin) with ayahuasca (and other psychedelics) that evaluate antidepressant effects in clinical populations have not been published to date. In healthy individuals, studies involving the concomitant administration of ketanserin with psilocybin [149], LSD [110], and ayahuasca [109] have shown that this drug blocks most of the subjective effects of these compounds. 

On the other hand, we do have preliminary evidence for the antidepressant effect of psilocybin, which is thought to be much more specific in its central nervous system (CNS) modulation effects by acting primarily and mostly on 5-HT_1A/2A/2C_ receptors [150,151]. In a first analysis, this would indicate that these receptors’ agonism is sufficient for the antidepressant effects to occur, but these investigations where psilocybin was administered to depressed patients also provided psychological support, which was not given in antidepressant effects investigations with ayahuasca and must account at least partially for the results reported. Furthermore, to the best of our knowledge there are no available drugs to specifically activate 5-HT_2A_ receptors that could be used to verify the extent to which they participate in the possible therapeutic effects of ayahuasca and other psychedelics. 

Another relevant serotoninergic target of ayahuasca’s effects is the 5-HT_2C_ Gq/G11 protein-coupled receptor. The 5-HT_2C_ receptors are abundant in choroid plexus epithelial cells and parvalbumin GABAergic neurons in the prelimbic PFC but are also expressed in many other limbic, cortical, and basal ganglia brain regions [114]. These receptors are intracellular messengers via phospholipase C, which triggers cell-signaling pathways related to the transcription of immediate early genes (IEGs) [114]. Being from the same family of receptors as 5-HT_2A_, they are also involved in IP3 and DAG intracellular accumulation via Gq/G11-protein signaling, ERK signaling, and arachidonic acid release via phospholipase A2 [114,152]. Depending on the agonist binding, these receptors will modulate which signaling pathway is activated, with psychedelic drugs including LSD, 2,5-dimethoxy-4-iodoamphetamine (DOI), and bufotenin (closely related to DMT) preferably activating the arachidonic acid/phospholipase A2 pathway [153].

Both DMT and harmine can bind at 5-HT_2C_ receptors with low affinity [140]. Although several classes of antidepressants modulate 5-HT_2C_ receptors, there is a need for further research to define their role in depression. Preclinical studies have provided conflicting evidence with regard to the agonism and antagonism at these receptors, as both types of modulation have resulted in antidepressant-like effects in preclinical investigations [154,155,156].

### 6.2. Glutamatergic System

The glutamatergic system is the main excitatory neurotransmitter system within the brain and is composed of several metabotropic glutamate G protein-coupled receptors (mGlusRs) and ionotropic glutamate receptors (iGluRs) [157]. Of special relation to ayahuasca’s effects are the AMPA receptors, ionotropic receptors distributed widely within the CNS, being found in the hippocampus, outer layer of the cortex, basal ganglia, lateral septum, amygdala, cerebellum, thalamus, and brain stem depending on the subunits expressed [158]. The interplay between 5-HT and AMPA receptors is thought to have an important role in the antidepressant and neuroplastic effects of ayahuasca, other psychedelics, and ketamine [159,160].

In this regard, DMT binding at 5-HT_2A_ receptors seems to modulate glutamatergic activity [100,161,162,163], which results in the activation of AMPA receptors. This in turn leads to the expression of BDNF, a ligand to tyrosine receptor Kinase B (TrkB) receptors which promotes the activation of the mechanistic target of the rapamycin complex-1 (mTORC1) pathway [160,164]. This mechanism seems to induce neuronal synaptic and structural plasticity [160,165] and could be a possible explanation for psychedelics' long-lasting therapeutic effects. As BDNF can facilitate glutamate release, this pathway results in sustained activation [166]. Furthermore, the activation of 5-HT_1A_ receptors located in the medial PFC has also been linked to the stimulation of the same pathway, which implicates not only DMT but also β-carbolines as protagonists in the neuroplastic effects of ayahuasca [159].

There are also further interactions between serotoninergic and glutamatergic systems that have been reported. Metabotropic glutamatergic receptors 2/3 (mGluR2/3) are modulated in the presence of 5-HT_2A_ agonists, and there is evidence that the 5-HT_2A_-mGluR2 heterodimer receptor is related to the effects of serotonergic psychedelics [56]. Preclinical studies have also demonstrated that concomitant administration of a 5-HT_2C_ receptor antagonist with DMT modestly attenuated DMT’s effects, while a mGluR2/3 receptor agonist potently blocked some of DMT’s effects and an antagonist at the same receptors facilitated DMT’s effects [56,167,168]. Furthermore, mGluR2 knockout mice had a greatly attenuated head twitch after DOI administration, which implicates these receptors in the effects of serotoninergic psychedelics [56]. Lastly, there is a study that implicated NMDA glutamatergic receptors as modulators of DMT’s effects. In this investigation, the effects of phencyclidine, an NMDA antagonist, were partially blocked by DMT administration [169]. However, the relationship of the above-cited interactions to the treatment of depression still warrants further research.

### 6.3. Dopaminergic System

Dopamine is a catecholamine related to multiple cognitive and behavioral functions, such as the reward system and working memory [170,171]. Dopaminergic receptors can be divided into two families, D1-like and D2-like receptors, which are coupled to stimulatory and inhibitory G proteins, respectively [172,173,174]. The D1-like receptor signaling pathway leads to the activation of adenylyl cyclase (AC), which promotes increased levels of cAMP, a second messenger that activates the protein kinase A. This pathway results in enhanced dopamine-activated phosphoprotein with 32kD (DARPP-32) expression and inhibits the protein phosphatase 1, responsible for the dephosphorylation of multiple cell proteins [174]. On the other hand, D2-like receptors inhibit AC expression, downregulating the D1 receptor signaling path [172].

The dopaminergic system has three main pathways. The substantia nigra projects neurons to the striatum, and this is the nigrostriatal pathway, involved in motor behavior [175]. Inputs from the ventral tegmental area (VTA) to the prefrontal cortex compose the mesocortical pathway, related to cognitive functions [176], and the VTA to NAc projections form the mesolimbic pathway, commonly referred to as the reward system [177]. Although depression could result in altered functioning in all dopaminergic pathways [178], mesolimbic projections can be directly linked to anhedonia, commonly observed in studies of depressive symptoms [20,179]. As for the mesocortical pathway, impaired functioning can result in the alterations in motivational behavior usually present in depressed individuals [176].

Ayahuasca modulation of the dopaminergic system may be primarily caused by β-carbolines’ ability to inhibit MAO-A [180], and, although β-carbolines do not seem to possess an affinity for dopaminergic receptors [99], the increase in the availability of monoamines caused by this inhibition can affect dopaminergic signaling, in a mechanism similar to that of the first antidepressants, the MAO inhibitors [181]. A study by Brierley and Davidson [57] demonstrated that harmine increases dopamine levels on the surface of the NAc. In this study, a comparison with moclobemide (a reversible, selective MAO-A inhibitor) did not cause the same effect, suggesting that the increase in dopamine concentrations in NAc may be related to its modulation by 5-HT_2A_ receptors, a mechanism of action still little explored [57]. Even though ayahuasca components are not effective ligands at dopamine receptors [99], DMT could interfere with dopaminergic signaling through sigma-1 receptors since these receptors can be expressed on dopaminergic neurons [175]. Furthermore, DMT has been shown to cause dopamine release from presynaptic stores, possibly related to modulation in dopamine turnover and striatal synthesis, affecting endogenous levels of dopamine metabolites [104].

Ayahuasca administration to rats also resulted in enhanced amygdala DA concentrations [182] and treating animals with β-carbolines can improve depressive-like symptoms caused by chronic unpredictable stress [20]. Additionally, D2 receptors can form heterodimers with 5-HT_2A_ receptors [183]. While psychedelics can increase excitability in cortical structures [161], the activation of D1-like receptors by an agonist is able to prevent DOI-driven excitability in rat brain slices, through cAMP signaling [184]. It is possible then that D2 receptors can play a role in enabling psychedelic effects by reducing cAMP signaling in the PFC. Furthermore, activation of D2 receptors in the PFC seems to improve depressive-like behavior in mice submitted to the tail suspension test [176]. 

The psychedelics’ effects on NAc activity are still not well understood, but LSD administration to rats resulted in increased c-Fos expression in the NAc [185], and ayahuasca and psilocybin intake increased the activity in the NAc of volunteers [40,186]. Since D1 and D2 activity result in opposite effects, it is possible that psychedelics might promote a preferential D2 receptor activation through modulation of cortical inputs to the NAc, consequently reducing the expression of the cAMP response element-binding protein (CREB).

### 6.4. Endocannabinoid System

The endocannabinoid receptors CB1 and CB2 are class A G protein-coupled receptors (GPCRs) that are widely distributed throughout the human body and are believed to be important in regulating endocrine, neurotransmission, neuroprotective, developmental, and immune processes [187,188,189]. These receptors are primarily involved in retrograde neurotransmission signaling and are thought to be important protagonists in the regulation and modulation of neuronal excitability and signal transmission [190]. CB1 receptor agonists inhibit forskolin-stimulated adenylyl cyclase, negatively modulate calcium channels (N-, P-, and Q-type), and stimulate potassium intracellular influx [189,191,192]. The CB2 receptor’s intracellular pathways include cyclooxygenase-2 (COX-2), mitogen-activated protein kinase and phosphoinositide 3-kinase pathways induction, and inhibition of adenylyl cyclase [193,194,195]. CB2 receptors are found in particularly high amounts in the immune system, and thus may provide a viable pathway to positively modulate inflammatory processes within the CNS which may be related to depressive symptoms [196]. Besides physiological functions, this system is also implicated in mood regulation, emotion processing and recognition, cognitive processes such as memory and learning, pain, and other homeostatic functions [188,197]. The most studied endocannabinoids which modulate this system are anandamide (AEA) and 2-arachidonoyl glycerol (2-AG). Both substances are thought to be important modulators of the biological processes cited above, within, and outside the CNS [198]. 

Preclinical evidence has shown that 5-HT_2A_ receptor activation is linked to 2-AG secretion [199,200] and that stereotypic behavior induced by 5-HT_2A_ receptor agonism is inhibited by the presence of CB1 receptor antagonists [201]. Furthermore, evidence supports the presence of heterodimer CB1+5-HT_2A_ receptors in brain regions involved in memory formation [202]. Although preliminary, there is evidence that this system is modulated by ayahuasca ingestion, albeit indirectly through changes in circulating levels of AEA and 2-AG [35,59]. The first study showed a decrease in anandamide and a slight increase in 2-AG plasma levels 4 hours after ayahuasca intake by one healthy volunteer [59]. The second showed acutely evaluated changes in AEA and 2-AG in nine healthy volunteers and five volunteers with social anxiety disorder (SAD) from two randomized, placebo-controlled trials after a single ayahuasca administration [35]. This preliminary evidence pointed to an increase (90 minutes after ayahuasca administration) followed by a decrease (240 minutes after ayahuasca administration) in the levels of AEA in SAD patients, with no significant changes in healthy volunteers. Nevertheless, the limited samples in RCTs to date preclude solid conclusions in regard to the modulation of the endocannabinoid system by ayahuasca and its possible role in the therapeutic effects of the brew.

### 6.5. Sigma-1 Receptors

The sigma-1 receptor is distributed widely throughout the human body within the CNS and vital organs and is believed to have a considerable role in the etiology of depression and other diseases [203,204,205]. These receptors are localized between the endoplasmic reticulum (ER) and mitochondria, and agonists at these receptors cause them to disassociate from ER chaperones which transforms them into chaperones to IP3 receptors [104]. The sigma-1 receptors are involved in important molecular biological processes such as calcium signaling from ER to mitochondria, regulation of the citric acid cycle and ATP production, ion channel regulation, cell survival, and the proliferation and potentiation of NMDA receptors [60,104,206]. 

Regarding the effects of ayahuasca, DMT is believed to be an agonist at sigma-1 receptors and its action has been related to the modulation of the immune system and neuroprotective effect against hypoxia [63,100,207,208]. DMT’s activation of these receptors has also been proposed to be a possible therapeutic route for the extinction of traumatic memories, which may be beneficial to the treatment of post-traumatic stress disorder but also aversive memories in depression [209]. Furthermore, a recent in vitro investigation has shown that treatment of neural stem cells with DMT increased neurogenic processes (e.g., proliferation, migration, and cell differentiation), resulting in increased neuronal and glial populations and better performance in episodic memory tasks [210]. These effects were prevented when treatment was combined with a sigma-1 receptor antagonist, but not when it was combined with 5-HT_1A/2A_ receptor antagonists, suggesting that the neurogenic enhancement promoted by DMT might not be dependent on serotonergic signaling [210]. There is also a possible association between DMT’s effects on synaptic plasticity and increased BDNF expression and its effect at sigma-1 receptors [211]. In fact, the combination of DMT's effects on serotoninergic and sigma-1 receptors seems to imbue this substance with a singular pharmacological profile, resulting in unique capabilities of neuroprotection and immune modulation [100,208]. To the best of our knowledge, no specific role of β-carbolines at these receptors has been reported to date. 

### 6.6. Neuroendocrine System

The etiology of depression has been related to neuroendocrine changes for many decades. Stress-related changes in HPA axis function which result in increased hypothalamic corticotropin-releasing hormone (CRH) and consequently cortisol secretion have consistently been associated with the emergence of depressive symptoms, although it is not yet understood if this contributes as a cause to depressive symptoms or is an epiphenomenon [212,213]. Preclinical evidence has indicated that antidepressant medications facilitate the negative feedback by endogenous glucocorticoids, which in turn reduces HPA axis activity [214]. Dysfunctions in thyroxine (T_4_), thyroid-stimulating hormone (TSH), and growth hormone (GH) secretion have also been reported in depressive patients and correlated with emergence of depressive symptoms [215,216,217,218,219]. It is also known that many receptors influenced by ayahuasca are expressed in the hypothalamus, including 5-HT_1A/2A/2C_ and sigma-1 receptors (reviewed in Schindler et al., 2018 [220]). Furthermore, preclinical evidence has previously shown that 5-HT_1A/2A_ signaling is involved in modulating CRH [221], adrenocorticotropic hormone (ACTH) and corticosterone [222,223,224] release, while clinical evidence with LSD and psilocybin administration demonstrates increases in ACTH and cortisol after drug exposure [225,226,227].

Clinical trials with ayahuasca have shown it is capable of modulating neuroendocrine variables in healthy and depressed patients. Regarding healthy volunteers, ayahuasca administration (1.0 mg DMT/kg body weight) significantly increased prolactin and cortisol secretion when compared with baseline [1]. Another study related the administration of two ayahuasca doses (0.75 mg DMT/kg body weight) 4 hours apart with significant increases in serum prolactin, GH, and cortisol when compared with placebo [84]. In depressive patients with hypocortisolemia, a normalization of salivary cortisol levels 48 hours after a single ayahuasca administration (0.36 mg DMT/kg body weight), was observed, although no significant changes were reported for plasma cortisol levels [69].

### 6.7. Other Molecular Targets/Systems

DMT interacts with other proteins implicated in monoaminergic neurotransmission including SERT and neuronal VMAT2 [60,98,100]. DMT is a substrate for these transporters, a mechanism which is thought to be responsible for gathering sufficient vesicular concentrations of the alkaloid (where it can remain stored for at least 1 week) for it to become endogenously active, supposedly at sigma-1 receptors and TAAR1s [104]. DMT’s agonism (and that of other psychedelics including LSD, DOI and 5-MeO-DMT) at TAAR-1s has been shown to activate adenylyl cyclase and promote cAMP accumulation, although there is still no research regarding the possible role (if any) of this in the alkaloid’s effects [104,211]. These accumulation and storage mechanisms are thought to be related to the influence DMT (and thus ayahuasca) has on the CNS [104].

With regard to β-carbolines, harmine demonstrates a high affinity for tyrosine-phosphorylation-regulated protein kinase of dual specificity 1A (DYRK1A) and moderate affinity for and imidazoline I2 receptors [228,229,230]. The ability of harmine to inhibit intracellular protein aggregation has also been previously observed alongside its antioxidant potential [231]. Furthermore, harmine has also been found to influence the basal amygdala and projection neurons through their modulation of GABAergic neurons and its low-intensity agonism to GABA-A receptors in rats [232]. Harmaline and harmine seem to have an antagonistic effect on alpha-1 adrenergic receptors in a non-competitive manner [233] and possible acetylcholinesterase inhibition properties [160]. Furthermore, β-carbolines have also been shown to stimulate adult neurogenesis in vitro, although the exact mechanism by which they achieve this has not yet been fully elucidated [108].

Concerning ayahuasca as a whole, there is one report that described reductions in C-reactive protein in healthy controls and depressed patients alike, with this reduction correlated with suppression of depressive symptoms in patients, although there were no significant results regarding interleukin-6 [36]. C-reactive protein is a biomarker primarily produced by the liver that has been related to many pro-inflammatory processes within the body, including the production of other pro-inflammatory biomarkers such as interleukin-6 and tumor necrosis factor-α [234]. Reductions in the levels of such biomarkers are related to reductions in the inflammatory response and are hypothesized to be beneficial for depressed patients, especially those with an augmented inflammatory state [235,236]. Nevertheless, these and other inflammatory biomarkers have not yet been found to be reliable as indicators of response to rapid-acting antidepressants (such as psychedelics and ketamine), and the mechanism by which ayahuasca modulates their levels is not fully understood [75,237]. In fact, all these ayahuasca interactions have not been thoroughly investigated and it is yet to be shown how much they contribute to the psychoactive and possible therapeutic effects of ayahuasca in humans.

## 7. Final Remarks

As evidenced in this review, the psychoactive and possible therapeutic effects of ayahuasca cannot be explained by a single receptor or neurotransmitter system. Given the complex combination of alkaloids contained in ayahuasca, the physiological and consequently mental effects of the brew probably arise from the modulation of multiple targets at once, in an intricate molecular symphony that gives ayahuasca a difficult but unique pharmacological profile. Preclinical and clinical studies that focus on the effects of its separate substances will continue to help unfold the effects of particular molecular targets, but ultimately future investigations should focus on researching how these effects interact with each other to fully elucidate ayahuasca’s possible therapeutic potential.

## Figures and Tables

**Table 1:** Clinical trials that investigated ayahuasca’s potential for the treatment of depression and related research.

Clinical Trials Investigating the Antidepressant Effects of Ayahuasca				
Reference	Design	Sample	Main Measures	Main Findings
Osório et al., 2015 ^1^ [37]	Preliminary Open-Label Study	Six Volunteers	HAM-D, MADRS, BPRS and YMRS	Reduction of 82% in depressive symptoms up to 21 days after administration
Sanches et al., 2016 ^2^ [40]	Open-Label	Seventeen volunteers	HAM-D, MADRS, BPRS, CADSS and YMRS	Reduction in depressive symptoms up to 21 days after administration, increased blood flow in the left nucleus accumbens, right insula and left subgenual area
Galvão et al., 2018 [69]	Randomized, Placebo-Controlled Trial	Seventy-one volunteers (twenty-eight of whom ingested ayahuasca)	HAM-D, MADRS and salivary cortisol	Normalization of cortisol levels in saliva 48 hours after administration without correlation with reduction in depressive symptoms
Palhano-Fontes et al., 2019 ^3^ [38]		Twenty-nine volunteers (fourteen of whom ingested ayahuasca)	HAM-D, MADRS, BPRS, CADSS, HRS, MEQ30 and YMRS	Reduction in depressive symptoms up to 7 days after administration
Zeifman et al., 2019 [70]			MADRS and MADRS-SI	Non-significant reduction in suicidal ideation
de Almeida et al., 2019 [71]		Seventy-three volunteers (twenty-eight of whom ingested ayahuasca)	HAM-D, MADRS and plasma BDNF	Higher plasma BDNF concentrations in the ayahuasca group 48 hours after administration when compared with placebo. Increases in BDNF correlated with reduction in depressive symptoms
Galvão-Coelho et al., 2020 [36]			HAM-D, MADRS, C-reactive protein and interleukin-6	Reduction in C-reactive protein levels correlated with reduction in depressive symptoms

BDNF: Brain-Derived Neurotrophic Factor; BPRS: Brief Psychiatric Rating Scale; CADDS: Dissociative States Rating Scale; Administered by the Clinician; HAM-D: Hamilton Depression Rating Scale; HRS: Hallucinogen Rating Scale; MADRS: Montgomery–Asberg Depression Scale; MADRS-SI: Montgomery-Asberg Depression Scale suicidal intent subscale; MEQ30: Mystical Experiences Questionnaire; YMRS: Clinician-Administered Dissociative States Rating Scale. ^1^ Preliminary study; ^2^ Definitive study including patients from the preliminary study; ^3^ Original study. All other studies that share the same design were derived from this experiment (randomized controlled trial).

**Table 2:** Ayahuasca’s alkaloid concentration reported in studies with at least 8 different samples.

	Reported Concentrations (mg/mL)				Number of Samples Analyzed
Reference	DMT	Harmine	Harmaline	THH	
McKenna et al., 1984 [5]	0.13–0.30	0.15–0.34	0–0.20	0.05–0.80	8
Callaway, 2005 [78]	0–14.15	0.45–22.85	0–0.90	0.48–23.8	29
Santos et al., 2017 [79]	0.30–36.70 ^1^				20
Souza et al., 2019 [80]	0.62–3.40	4.14–18.16	0.40–3.92	4.02–30.88	38
Santos et al., 2020 [81]	0.10–3.12	0.11–7.11	0.01–0.94	0.09–3.05	33
Kaasik et al., 2021 [82]	0–2.68	0.06v4.44	0–0.33	0.01–3.87	102

DMT: N,N-Dimethyltryptamine; THH: Tetrahydroharmine. ^1^ Individual values for each analysis not specified by the authors. The values presented represent the minimum and maximum of all analyses.

**Table 3:** Ayahuasca’s alkaloid concentrations and dosages from clinical trials with ayahuasca.

Reference	Standard DMT Dose (mg/kg)	Ayahuasca Alkaloid Concentration (mg/mL)				Mean Dosage per Mean Body Weight of Participants (mg)			
		DMT	Harmine	Harmaline	THH	DMT	Harmine	Harmaline	THH
Riba et al., 2001 ^1^ [83]	0.50	0.53	0.90	0.06	0.72	35.75	60.65	4.12	48.75
	0.75					53.63	90.97	6.14	74.42
	1.00					71.50	121.28	8.24	97.50
Riba et al., 2003 ^2^ [11]	0.60					39.80	67.40	4.60	54.20
	0.85					57.40	95.80	6.50	77.00
Riba et al., 2006 [39]	1.00					66.80	113.31	7.69	91.09
dos Santos et al., 2011 [1]	1.00					67.00	113.65	7.72	91.37
dos Santos et al., 2012 ^3^ [84]	0.75					52.26	88.64	6.02	71.26
Sanches et al., 2016 [40]	-	0.80	0.21	ND	NA	128.00	33.50	-	-
Palhano-Fontes et al., 2019 [38]	-	0.36	1.86	0.24	1.20	25.77	133.15	19.32	85.00
Rocha et al., 2021b [85]	-	0.67	0.87	0.27	0.38	109.36	79.92	7.38	50.71
dos Santos et al., 2021 [86]	-	0.68	0.52	0.14	0.62	87.44	66.87	18.00	79.73
Total mean	0.81	0.61	0.87	0.18	0.73	66.23	88.76	8.70	74.64

DMT: N, N-Dimethyltryptamine; NA: Not Analyzed; ND: Not Detected; THH: Tetrahydroharmine. ^1^ Administration of three different doses; ^2^ Administration of two different doses; ^3^ Administration of two equal doses in four hours apart.
